# Estimation of the number of histological diagnosis for IgG4-related kidney disease referred to the data obtained from the Japan Renal Biopsy Registry (J-RBR) questionnaire and cases reported in the Japanese Society of Nephrology Meetings

**DOI:** 10.1007/s10157-016-1260-1

**Published:** 2016-03-25

**Authors:** Hitoshi Nakashima, Mitsuhiro Kawano, Takako Saeki, Yoshifumi Ubara, Satoshi Hisano, Michio Nagata, Yoh Zen, Motoko Yanagita, Yutaka Yamaguchi, Shinichi Nishi, Takao Saito

**Affiliations:** 1 Division of Nephrology and Rheumatology, Department of Internal Medicine, Faculty of Medicine, Fukuoka University, 7-45-1 Nanakuma, Jonan-ku, Fukuoka, 814-0180 Japan; 2 Division of Rheumatology, Department of Internal Medicine, Kanazawa University Hospital, Kanazawa, Japan; 3 Department of Internal Medicine, Nagaoka Red Cross Hospital, Nagaoka, Japan; 4 Nephrology Center and Okinaka Memorial Institute, Toranomon Hospital, Tokyo, Japan; 5 Department of Pathology, Faculty of Medicine, Fukuoka University, Fukuoka, Japan; 6 Department of Kidney and Vascular Pathology, Graduate School of Comprehensive Human Sciences, University of Tsukuba, Tsukuba, Japan; 7 Department of Diagnostic Pathology, Kobe University Graduate School of Medicine, Kobe, Japan; 8 Department of Nephrology, Kyoto University Graduate School of Medicine, Kyoto, Japan; 9 Yamaguchi’s Pathology Laboratory, Chiba, Japan; 10 Division of Nephrology and Kidney Center, Kobe University Graduate School of Medicine, Kobe, Japan

**Keywords:** IgG4-RKD, Histological diagnosis, Geographic distribution

## Abstract

**Background:**

More than 2 years have passed since the proposal of the diagnostic criteria for IgG4-related kidney disease (IgG4-RKD). The aim of this study was to estimate the number of histological diagnosis for IgG4-RKD throughout Japan and to clarify the regional distribution of the development of this disease.

**Methods:**

A questionnaire was supplied to 140 research facilities registered in the Japan Renal Biopsy Registry (J-RBR). The items of the questionnaire were the total number of renal biopsies performed and the number of cases diagnosed as IgG4-RKD in 2012 and 2013 at each facility. Age, sex, and diagnosis category were also included for the IgG4-RKD cases. The geographic distribution of the disease development was evaluated using clinical case reports presented at the Eastern/Western regional meeting of the Japanese Society of Nephrology during the 15 years following 2001.

**Results:**

Forty-seven facilities completed the questionnaire, resulting in a collection rate of 34 %. The total numbers of renal biopsies in 2012 and 2013 were 3387 and 3591, respectively. Forty-seven of these cases (24 in 2012 and 23 in 2013) were diagnosed as IgG4-RKD. The frequency of development of IgG4-RKD per one million over 40-year-old individuals during these 15 years varied between 0.9 and 3.1, depending on Japanese geographic region of Japan.

**Conclusion:**

The results of the present survey indicate that the number of diagnosis for IgG4-RKD is approximately 130 cases per year throughout Japan, and no regional differences in disease frequency appear to exist.

## Introduction

**Fig. 1:**
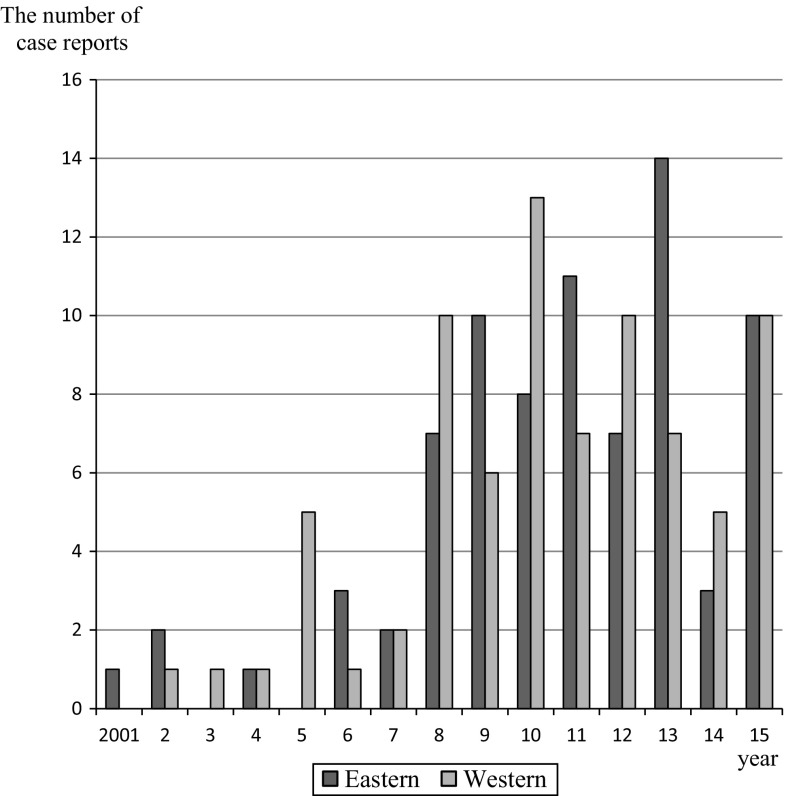
The distribution of clinical case reports for IgG4-related kidney disease presented at the Eastern/Western regional meeting of the JSN during the recent 15 years

The accumulation of clinical cases with extra-pancreatic lesions associated with autoimmune pancreatitis (AIP) led to the establishment of IgG4-related disease (IgG4-RD) as a new clinical disease. Although the renal disorder was first described in relation to AIP, it is now considered to be a characteristic condition within the category of IgG4-RD [1, 2]. In fact, the number of case reports for tubulointerstitial nephritis associated with IgG4-RD presented at the Eastern/Western regional meeting of the Japanese Society of Nephrology (JSN) has gradually increased since 2001 (Fig. 1). Therefore, the “IgG4-related Kidney Disease” Working Group (IgG4-RKD WG) was assembled under the JSN’s Committee for Standardized Pathological Kidney Diagnosis and proposed diagnostic criteria and an algorithm for the diagnosis of IgG4-RKD in 2011 [3]. The diagnostic criteria are based on clinical and serological features, imaging features, histology, and other organ involvements. Histology, including immunostaining, is the most important item, and a definite diagnosis cannot be established without appropriate histological findings in the kidney or other organs. The diagnosis is classified into three categories: definite, probable, and possible.

More than 4 years have passed since the WG proposed the diagnostic criteria for IgG4-RKD, and, with the approval of the ethical board of the JSN (approval number: 7), the WG has performed a multi-center retrospective study of clinicopathological features of IgG4-RKD. The present study aimed to determine how frequently the diagnosis of IgG4-RKD was made throughout Japan. To address this question, we designed a brief online frequency questionnaire to be administered to Japanese nephrologists.

## Materials and methods

The questionnaire was supplied to 140 research facilities registered as secondary facilities in the Japan Renal Biopsy Registry (J-RBR), which is a nationwide, web-based, prospective registry system designed to record pathological, clinical, and laboratory data about renal biopsies performed in Japan [4]. The questionnaire items included assessments of the total number of renal biopsies and the number of cases diagnosed as IgG4-RKD in 2013 and 2014 at each facility. Hereafter, we call this data the “2-year material.” The protocol for this study was approved by the ethics committee of Fukuoka University Hospital in accordance with the guideline on epidemiological research from Japan’s Ministry of Health, Labor, and Welfare (approval number: 15-1-16). In addition to the questionnaire administration, age, sex, and diagnosis category were sampled from the case reports presented at the JSN’s Eastern/Western regional meeting in the 15 years following 2001. Hereafter, we call this data the “15-year material.” Furthermore, to determine the geographic distribution of the development of IgG4-RKD in Japan, we calculated the numbers of IgG4-RKD cases from every region of Japan using these two types of materials. Every prefecture population data was referred to “Population by five-year age group and prefecture” in Japan statistical yearbook 2013 offered by Statics Japan (http://www.stat.go.jp/).

Continuous variables are shown as mean values ± standard deviation (SD). Quantitative variables were compared using Student’s *t* test or Chi square test. The frequency of IgG4-RKD in the 15-year material among the different regions was compared using one-way analysis of variance (ANOVA) and Kruskal–Wallis test. *p* values of < 0.05 (obtained by two-tailed testing) were considered to indicate statistical significance.

## Results

**Fig. 2:**
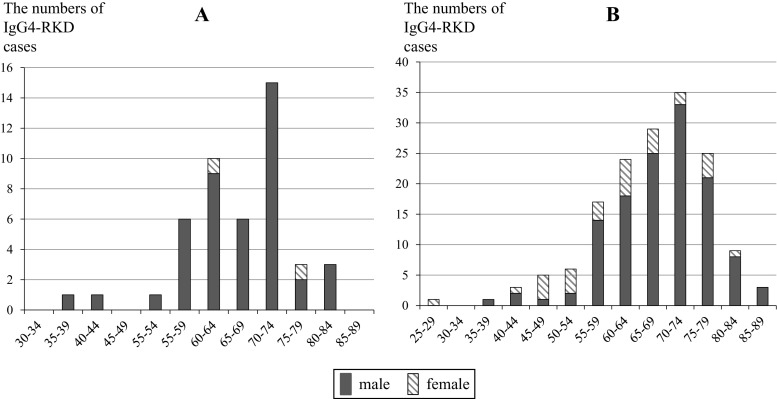
Distribution of age ranges and sex in 2-year material (**a**) and 15-year material (**b**)

A total of 47 nephrologists (44 physicians and 3 pediatricians) affiliated with J-RBR-registered facilities completed the questionnaire (collection rate: 34 %). The total numbers of renal biopsies performed in these facilities in 2012 and 2013 were 3387 and 3591, respectively. Among these patients, 47 (24 in 2012 and 23 in 2013) were diagnosed with IgG4-RKD. This resulted in a rate of 6.7 IgG4-RKD cases per 1000 renal biopsies. Patient informations of age, sex, and diagnosis category were obtained besides one patient. The ages of the 46 patients ranged from 38 to 81 years, and the average and median values were 66.8 ± 9.3 and 67.5 years, respectively. The ratio of men to women was 44:2 (Fig. 2a). Thirty-five diagnoses were categorized as definite (76.1 %), 4 as probable (8.7 %), 6 as possible (13 %), and 1 as unknown.

One hundred fifty-eight clinical cases that may have corresponded to IgG4-RKD were sampled from the 15-year material. The numbers of cases reported in the Eastern and Western regional meetings appeared to be comparable (Fig. 1). The ages of the patients ranged from 25 to 86 years, and the average and median values were 66.9 ± 10.4 and 68.0 years, respectively. The ratio of male to female patients was 128:30 (Fig. 2b). There was a significant sex difference for the development of IgG4-RKD, and the average ages also differed significantly between males and females (68.4 ± 9.2 vs 60.4 ± 12.9 years). One hundred-twenty diagnoses were categorized as definite (76.0 %), 13 as probable (8.2 %), and 25 as possible (15.8 %). These category proportions were almost equal to those obtained from the 2-year material.

To determine the geographic distribution of the development of this disease in Japan, every case was assigned to a certain region based on the location of the corresponding hospital. The diagnosis frequencies per thousand renal biopsies using the 2-year material and the development frequencies per one million over 40-year-old individuals using the 15-year material were calculated in every district of Japan. Two cases younger than 40-year-old were excluded in this analysis to compare with the middle-age and elderly population size and the total number of cases was 156 in Table 1. For the histological diagnosis frequency in 2-year material the number of cases was not enough to evaluate the significant difference among districts. Concerning the development frequency in 15-year material the maximal frequency of 3.1 was obtained in the Hokuriku-Koshinetsu region, and the minimal frequency of 0.9 in the Tohoku regions. When the data were examined using One-way ANOVA and Kruskal–Wallis test, however, there were no significant differences among the frequencies of these areas (*p* = 0.72).

**Table 1:** Regional variation in the development of IgG4-RKD

District	2-year material			15-year material		
	Cases	Total biopsies	A	Cases	Population ≥40 years old ×10^3^	B
Hokkaido	3	197	15.2	6	3387	1.8
Tohoku	10	1246	8.0	5	5642	0.9
Kanto	4	1063	3.8	61	24,937	2.4
Hokuriku-Koshinetsu	13	860	15.1	16	5132	3.1
Tokai	3	1304	2.3	13	8796	1.5
Kinki	7	532	13.2	27	12,332	2.2
Chugoku	2	628	3.2	8	4549	1.8
Shikoku	2	414	4.8	5	2442	2.0
Kyushu-Okinawa	3	734	4.1	15	8606	1.7
Total	47	6978	6.7	156	75,823	2.1

*A* The diagnosis frequencies per thousand renal biopsies, *B* The development frequencies per million among older than 40-year-old individuals

## Discussion

IgG4-RD is characterized by consistent pathological features, including tumefactive lesions and dense lymphoplasmacytic infiltrate rich in IgG4-positive plasma cells with fibrosis across a wide range of organ systems [1, 2]. IgG4-RD was first designated in Japan, and comprehensive diagnostic criteria were established, in 2012 [5]. Despite insufficient epidemiologic research, Umehara et al. estimated that 2.8–10.8/million population/year, with 336–1300 patients are newly diagnosed per year throughout Japan based on the numbers of visiting patients of two university hospitals in the Ishikawa prefecture [1]. In our daily medical practice, patients with IgG4-RKD are encountered significantly less often than those with IgG4-related dacryoadenitis and sialadenitis, which is representative of IgG4-RD. According to the results of the 2-year material, the histological diagnosis frequency for IgG4-RKD was 0.67 % of the total biopsied cases in 47 J-RBR-registered facilities. With regard to the number of native renal biopsies performed in Japan per year, the Research Group on Progressive Renal Disease from Japan’s Ministry of Health, Labor, and Welfare recently determined, using a questionnaire, that biopsies were performed in 17,000–21,000 and 20,000–22,000 cases in 2012 and 2013, respectively [ 6 ]. From these data, the number of histological diagnosis for IgG4-RKD can be roughly estimated to be 130 (114–147) cases per year throughout Japan. Most patients with IgG4-RKD have IgG4-related extra-renal lesions, with salivary glands, lacrimal glands, lymph nodes, and the pancreas being most frequently affected [3, 7–10]. According to diagnostic criteria, in the case of the qualifying requirement for histologic findings in extra-renal organs, characteristic renal radiologic findings were recommended to diagnose IgG4-RKD despite unperformed renal biopsies. Meanwhile in this study, the objects were biopsied cases and such particular cases were not included. Therefore, the obtained the number might be underestimated.

It was reported that IgG4-RKD is strikingly more predominant in men, and average patient age is about 65 years [3, 7–10]. The results of the 2-year material also indicated that elderly men were predominantly affected; thus, the average age was 66.8 ± 9.3 years, and 95 % of the patients newly diagnosed within 2 years were male. Although 2-year material and 15-year material were constituted with extremely rough data, these characteristics were the same as those obtained from the 15-year material. These numbers are apparently higher than those for other types of IgG4-RD, such as autoimmune pancreatitis [11] and IgG4-related dacryoadenitis and sialadenitis [12]. Although rapidly progressive glomerulonephritis (RPGN) also affects the elderly, its sex ratio is apparently different from that of IgG4-RKD. It was reported the ratio of male was 40.9 % of the RPGN patients diagnosed between 2002 and 2007 by the RPGN Registry Group [13]. According to the recently reported results of a questionnaire by the Research Group on Progressive Renal Disease from Japan’s Ministry of Health, Labor, and Welfare, the numbers of new patients with biopsy-proven RPGN in 2013 was 514, which corresponds to 5.4 % of the total cases with renal biopsy in medical training facilities authorized by the JSN [6]. The diagnosis of RPGN was supported by biopsy in 55.4 % of the patients, and the incidence of RPGN in 2013 throughout Japan was estimated to be 2100–2400. Renal amyloidosis is also an elderly onset renal disease. According to the data registered with the J-RBR from July 2007 to 2010, the development frequency of renal amyloidosis was 1.5 % of all biopsied cases (137/9439) [ 4, 14 ]. The frequency of these diseases increased together with age, 3.9 % in the case of those 60-year- old and older and 7.2 % in the cases of those 80-year-old and older [15]. Therefore, the number of histological diagnosis for IgG4-RKD is less than one-tenth that of RPGN, and less than one-half that of renal amyloidosis. General nephrologists seem to accept the practicality of these ratios.

The geographical distribution of the development of IgG4-RKD was evaluated using both “2-year” and “15-year” materials (Table 1). In 2-year material the numbers of cases in each district were so small that un-meaningful examination with the expectancy less than 5 was performed. Then every case was re-assigned to Eastern or Western Japan and Chi square test between them was performed. Finally it was revealed that there was no significant difference in diagnosis frequency between them. While for development frequency in 15-year material, the frequency in the Hokuriku-Koshinetsu region was 3.1 cases per one million over 40-year-old individuals, which is the largest value seen in Japan. Many researchers who have been interested in IgG4-RD since early stage are located in this region, and their highly accurate diagnoses might have contributed to this high frequency. Nevertheless, we found no significant differences among the numbers of regional variations in the development of IgG4-RKD. In this study both materials were rough and not available for close examination to reveal the significant differences, but showed that IgG4-RKD is found throughout Japan. Precise regional difference remains to be studied.

## Conclusions

In summary, although IgG4-RKD was recently recognized as a clinical entity, and applicable diagnostic criteria and an algorithm were proposed in 2011, epidemiological data are still insufficient. Based on the J-RBR questionnaire utilized in the present study, the number of histological diagnosis of IgG4-RKD in Japan is estimated to be approximately 130 cases per year. There was no significant difference in diagnosis frequency per thousand renal biopsies between Eastern and Western Japan. In addition, the evaluation of available clinical cases reported within the past 15 years shows no regional variations in the development of this disease. Accordingly, we should pay attention to IgG4-RKD as one of the important kidney disease in the elderly distributed all over Japan.

## Appendix

The following facilities responded to the questionnaire:

Hokkaido District

- Asahikawa Medical University Hospital (Division of Cardiology, Nephrology, Pulmonology, and Neurology, Department of Internal Medicine)
- Hokkaido University Graduate School of Medicine (Department of Medicine II)
- Hokkaido University Graduate School of Medicine (Department of Pediatrics).

Tohoku District

- Iwate Prefectural Central Hospital (Department of Nephrology)
- Japan Community Healthcare Organization, Sendai Hospital (Department of Nephrology)
- Tohoku University Hospital and affiliated hospitals (Department of Nephrology, Endocrinology, and Hypertension)
- Yamagata University School of Medicine (Department of Cardiology, Pulmonology, and Nephrology).

Kanto District

- Dokkyo Medical University Koshigaya Hospital (Department of Nephrology)
- National Center for Child Health and Development (Department of Nephrology and Rheumatology)
- Saitama Medical University, Saitama Medical Center (Department of Nephrology and Hypertension)
- Showa University Fujigaoka Hospital (Division of Nephrology)
- St. Marianna University School of Medicine (Division of Nephrology and Hypertension, Department of Internal Medicine)
- Tokyo Metropolitan Children’s Medical Center (Department of Nephrology)
- University of Tsukuba, Faculty of Medicine (Department of Nephrology).

Hokuriku-Koshinetsu District

- Nagaoka Red Cross Hospital (Department of Internal Medicine)
- Kanazawa Medical University School of Medicine (Division of Nephrology)
- Kanazawa University Hospital (Division of Rheumatology, Department of Internal Medicine)
- Niigata University Graduate School of Medical and Dental Sciences (Division of Clinical Nephrology and Rheumatology)
- Public Central Hospital of Matto-Ishikawa
- Shinshu University School of Medicine (Division of Nephrology)
- Toyama City Hospital (Department of Nephrology)
- Toyama Prefectural Central Hospital (Department of Internal Medicine)
- University of Fukui, Faculty of Medical Sciences (Division of Nephrology).

Tokai District

- Nagoya University Hospital (Department of Nephrology)
- Japan Community Healthcare Organization, Yokkaichi Hazu Medical Center (Department of Nephrology and Dialysis)
- Nagoya Kyoritsu Hospital (Department of Internal Medicine)
- Shizuoka General Hospital (Department of Nephrology).

Kinki District

- Kitano Hospital, the Tazukekofukai Medical Research Institute (Division of Nephrology and Dialysis)
- Kobe University Graduate School of Medicine (Division of Nephrology and Kidney Center)
- Kyoto Prefectural University School of Medicine (Division of Nephrology, Department of Medicine)
- National Cerebral and Cardiovascular Center (Division of Hypertension and Nephrology)
- Osaka University Graduate School of Medicine (Department of Geriatric Medicine and Nephrology)
- Wakayama Medical University (Department of Pediatrics).

Chugoku District

- Hiroshima University Hospital (Department of Nephrology)
- Kurashiki Central Hospital (Division of Nephrology)
- Mizushima Kyodo Hospital (Department of Internal Medicine)
- Okayama University Graduate School of Medicine, Dentistry, and Pharmaceutical Sciences (Department of Medicine and Clinical Science).

Shikoku District

- Kagawa University, Faculty of Medicine (Department of Cardiorenal and Cerebrovascular Medicine)
- Kochi University, Kochi Medical School (Department of Endocrinology, Metabolism, and Nephrology)
- Kochi University, Kochi Medical School (Department of Pediatrics)
- The University of Tokushima, Graduate School of Medicine (Department of Nephrology).

Kyushu District

- Fukuoka University School of Medicine (Division of Nephrology and Rheumatology, Department of Internal Medicine)
- Japanese Red Cross Fukuoka Hospital (Nephrology and Dialysis Center)
- Japanese Red Cross Fukuoka Hospital (Department of Pediatrics)
- Nagasaki University Hospital (Second Department of Internal Medicine)
- Saga University, Faculty of Medicine (Department of Cardiovascular and Renal Medicine)
- University of Occupational and Environmental Health (Second Department of Internal Medicine).
